# Antiplatelet Therapy in Breast Cancer Patients Using Hormonal Therapy: Myths, Evidence and Potentialities – Systematic Review

**DOI:** 10.5935/abc.20180138

**Published:** 2018-08

**Authors:** Andréa de Melo Leite, Ariane Vieira Scarlatelli Macedo, Antonio José Lagoeiro Jorge, Wolney de Andrade Martins

**Affiliations:** 1 Programa de Pós-graduação em Ciências Cardiovasculares da Universidade Federal Fluminense (UFF), Niterói, RJ - Brazil; 2 Rede D'Or São Luiz, Rio de Janeiro, RJ – Brazil; 3 Grupo Oncoclínicas do Brasil, Belo Horizonte, MG – Brazil

**Keywords:** Breast Neoplasms/drug therapy, Indicators of Morbidity and Mortality, Aspirin, Tamoxifen, Raloxilene Hydrochloride, Cardiovascular Diseases/prevention & control, Selective Estrogen Receptor Modulators

## Abstract

Breast cancer is the most frequently diagnosed tumor in women worldwide, with a significant impact on morbidity and mortality. Chemotherapy and hormone therapy have significantly reduced mortality; however, the adverse effects are significant. Aspirin has been incorporated into clinical practice for over 100 years at a low cost, making it particularly attractive as a potential agent in breast cancer prevention and as an adjunct treatment to endocrine therapy in the prophylaxis of cardiovascular complications. The objective of this study was to evaluate the role of aspirin in reducing the incidence of breast cancer and to evaluate the impact of its use on morbidity and mortality and reduction of cardiovascular events as adjuvant therapy during breast cancer treatment with selective estrogen receptor modulators. A systematic review was performed using the PRISMA methodology and PICO criteria, based on the MEDLINE, EMBASE and LILACS databases. The original articles of clinical trials, cohort, case-control studies and meta-analyses published from January 1998 to June 2017, were considered. Most studies showed an association between the use of selective estrogen receptor modulators and the increase in thromboembolic events. The studies suggest a protective effect of aspirin for cardiovascular events during its concomitant use with selective estrogen receptor modulators and in the prevention of breast cancer. This systematic review suggests that aspirin therapy combines the benefit of protection against cardiovascular events with the potential reduction in breast cancer risk, and that the evaluation of the benefits of the interaction of endocrine therapy with aspirin should be further investigated.

## Introduction

Breast cancer is the most frequently diagnosed tumor in women worldwide, with a significant impact on morbidity and mortality. According to the World Health Organization, it is estimated that more than 1.5 million new cases of breast cancer are annually diagnosed worldwide. Despite advances in treatment, breast cancer mortality is still high, with 570,000 deaths in 2015. The disease, recurrent or metastatic, remains incurable in most cases.^1^

Chemotherapy and hormone therapy have significantly reduced mortality, but their adverse effects are considerable. Endocrine therapy has revolutionized the treatment of breast cancer patients with positive Estrogen Receptor (ER), although there are cases that develop resistance to this therapy. An appropriate strategy would be the combination of Selective Estrogen Receptor Modulators (SERMs) or another hormonal class with other therapeutic agents, aiming at attaining a synergistic antitumor effect. The use of non-steroidal anti-inflammatory drugs (NSAIDs), including aspirin, has been associated with reduced risk of breast cancer.^2^^,^^3^ This therapy could also antagonize thrombogenic effects in women treated with tamoxifen.

The increasing number of breast cancer survivors is confronted with the shortage of information among clinicians on the subject.

The aim of the present study is to evaluate the role of aspirin in reducing the incidence of breast cancer and to evaluate the impact of its use in reducing cardiovascular events as an adjuvant therapy during the treatment of breast cancer with SERMs.

## Methods

This systematic review was carried out according to the Preferred Reporting Items for Systematic Reviews and Meta-Analyses (PRISMA) methodology.^4^ The study included original articles of clinical trials, cohort, case-control studies and meta-analyses published from January 1998 to June 2017, with full-texts in English, Spanish and Portuguese, obtained from the MEDLINE, EMBASE and LILACS databases. The research was performed using the following descriptors: (selective estrogen receptor modulators OR tamoxifen OR raloxifene hydrochloride OR toremifene) AND (platelet aggregation inhibitors OR aspirin) AND (cardiovascular disease) AND (breast CA).

This study was based on the PICO (acronym for Population, Intervention, Control and Outcome) criteria. The objective was to evaluate whether aspirin use implies in the reduction of events, especially cardiovascular events, in women with breast cancer using SERMs. The studies were selected according to the following criteria: use of SERMs in women with breast cancer; regular aspirin use; and evaluation of mortality, metastases, and adverse effects using SERMs and/or aspirin. Case reports, articles with other types of endocrine therapy, and animal experimental models were excluded.

**Figure 1:**
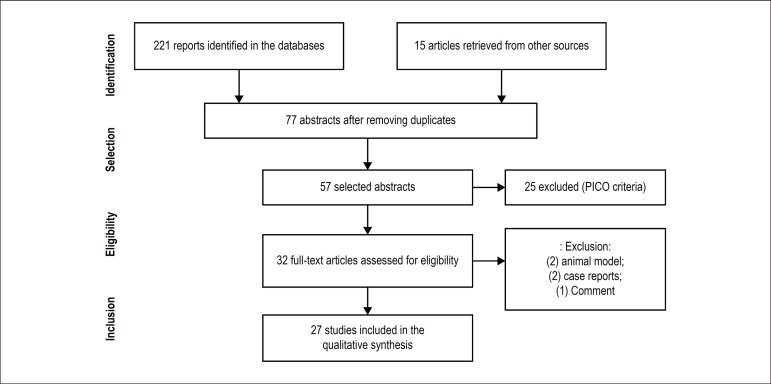
Flowchart of the evaluated studies.

A total of 221 abstracts met the search criteria and other 15 were manually retrieved. A total of 159 duplicated articles were eliminated and 77 abstracts were evaluated. Of these, 57 were selected for the review. We excluded 25 because they did not meet the previously established criteria, resulting in 32 full-text articles, which were evaluated in relation to their scientific quality. Five articles were excluded according to the inclusion/exclusion criteria. A total of 27 articles were analyzed, according to figure 1.

### Selective estrogen receptor modulators and reduction of morbidity and mortality in breast cancer

Most breast cancers have positive ER and three main drugs are being used for their treatment and/or prevention, namely: tamoxifen, raloxifene and toremifene. All of these agents are competitive inhibitors of estrogen binding to its receptors, and have mixed agonist and antagonist activity, depending on the target tissue.^5^ Tamoxifen is the most well-studied SERM and often the drug of choice for breast cancer treatment. Its mechanism of action involves tumor cell growth inhibition through competitive ER inhibition.^6^

The benefits of tamoxifen have been consolidated through the US Financial Service Task Force (USPSTF) meta-analysis.^7^ In comparison with placebo, the use of tamoxifen resulted in: reduced risk of invasive breast cancer (Relative Risk – RR = 0.70; 95% Confidence Interval – 95%CI: 0.59-0.82); reduction in the incidence of non-vertebral fractures (RR = 0.66, 95%CI: 0.45-0.98); and no difference in mortality from breast cancer or from all causes. On the other hand, a pro-coagulant effect is described when tamoxifen is added to chemotherapy – especially an increase in thromboembolic events.^8^^,^^9^

Raloxifene differs from tamoxifen because it does not stimulate endometrial tissue, although it exerts the same beneficial effects of tamoxifen on breast tissue. In preclinical studies, raloxifene has been shown to prevent the onset of new breast cancers, as well as prevent the growth of preexisting cancers.^10^ In the STAR (Study of Tamoxifen and Raloxifene) study,^11^ 19,747 women were randomized to receive 20 mg of tamoxifen or 60 mg/day of raloxifene for 5 years. The results showed that raloxifene had the same efficacy as tamoxifen in the prevention of breast cancer *in situ*, both with a 50% risk reduction (RR of 1.02, 95%CI: 0.82-1.28). However, raloxifene did not show protection against invasive types of breast cancer, whereas tamoxifen reduced its incidence by around 50%. It was observed that the group treated with raloxifene had an almost 30% reduction in thromboembolic events such as Deep Vein Thrombosis (DVT) and Pulmonary Embolism (PE) (RR = 0.70, 95%CI: 0.54-0.91). Both groups had the same incidence of cerebrovascular accident, myocardial infarction and fractures.

The MORE (Multiple Outcomes of Raloxifene Evaluation)^12^ study randomized 7,705 postmenopausal patients who had osteoporosis and had no history of breast or endometrial cancer for the use of placebo or 60 mg/day or 120 mg/day of raloxifene. After 4 years of follow-up, a 72% reduction of breast cancer risk was observed.^13^ In the CORE (Continuing Outcomes relevant to Evista) study,^14^ the patients were randomized to either raloxifene 60 mg/day or placebo. A 59% reduction (RR = 0.41, 95%CI: 0.24-0.71) was observed in the incidence of breast cancer and a decrease of 66% (RR = 0.34, 95%CI: 0.18-0.66 ) of ER-positive invasive breast cancer, when compared with the placebo group. When analyzing both studies together, the incidence of invasive breast cancer was reduced by 66% (RR = 0.34, 95% CI: 0.22-0.50) and, for ER-positive cases, 76% (RR = 0.24, 95%CI: 0.15-0.40), relative to the placebo group. No protection was observed against non-invasive cancers.

A significant reduction in the amount of microvessels in breast cancer was observed after treatment with raloxifene 60 mg/day for 28 days in postmenopausal women without previous endocrine treatment.^15^ There is evidence that the benefits of treatment with SERMs were seen not only during the 5 years of active treatment, as well as 5 years after the end of treatment, indicating a long-term effect on the prevention of breast cancer. Adverse effects, notably the thromboembolic events and endometrial cancer, should be considered when assessing the risk-benefit ratio for each patient.^16^

### Selective estrogen receptor modulators and thromboembolic events

A number of studies have demonstrated that the use of tamoxifen is associated with an increased rate of venous thromboembolic events (VTE) and that there is an additional procoagulant effect when tamoxifen is added to chemotherapy.^6^^,^^8^^,^^9^ Raloxifene is also associated with a higher risk of VTE, but with a lower incidence than tamoxifen. The NSABP (National Surgical Adjuvant Breast and Bowel Project) Tamoxifen Prevention Trial^9^ allocated 13,388 women at high risk of breast cancer to receive tamoxifen or placebo. The incidence of PE and DVT increased in women who received tamoxifen, especially in patients older than 50 years (RR for PE = 3.0, 95%CI: 1.1-11.2, RR for DVT = 1.6; 95%CI: 0.9-2.9). The IBIS-1 (International Breast Cancer Intervention Study)^8^ allocated 7,154 women at risk for breast cancer to receive tamoxifen or placebo. The use of tamoxifen was associated with an increased risk of developing VTE (Odds Ratio – OR = 2.1, 95%CI: 1.1-4.1). The risk of developing PTE or PE was significantly higher during the 5 years of active treatment with tamoxifen (RR of 2.3; 95%CI 1.4-3.9) but did not persist after its cessation.

A meta-analysis of seven trials and 30,023 patients, which compared outcomes in women with breast cancer assigned to treatment with tamoxifen or an aromatase inhibitor, found a higher rate of VTE in those receiving tamoxifen (2.8% vs. 1.6%).^16^ An analysis of 13 trials of the NSABP,^17^ which evaluated the risk of contralateral breast cancer in 20,878 women who received tamoxifen after primary treatment for this disease, found an increased risk of VTE with tamoxifen. The risks of PE, DVT and superficial phlebitis increased two to three-fold in patients treated with tamoxifen, and 11 to 15-fold in patients treated with tamoxifen plus chemotherapy.^18^ The STAR (Study of Tamoxifen and Raloxifene)^11^ study suggested a lower incidence of DVT and PE in women receiving raloxifene vs. those treated with tamoxifen. This study randomized 19,747 women at risk for breast cancer to raloxifene and tamoxifen use for 5 years.

### Selective estrogen receptor modulators and cerebrovascular accident

In the EBCTCG (Early Breast Cancer Trialists' Collaborative Group)^6^ meta-analysis, which compared 21,457 women to receive tamoxifen or placebo, there was an increase in cerebrovascular accident (CVA) rates, but without statistical significance. In a case-control study of 11,045 women with breast cancer, the risk of CVA was not increased by the use of tamoxifen.^19^ In a meta-analysis that evaluated the use of tamoxifen in primary or secondary prevention in 39,601 breast cancer patients, the frequency of ischemic CVA was higher in those who received tamoxifen than in the controls.^20^ Tamoxifen was associated with an increased risk of CVA, but with a low absolute risk.

In the RUTH (Raloxifene Use for The Heart) study,^21^ raloxifene was associated with an increased risk of fatal CVA when compared with placebo. The IBIS-1 study^22^ did not show statistical significance between the treatment groups (tamoxifen vs. placebo) regarding cerebrovascular or cardiovascular events. A sub-analysis of the MORE^12^ study suggested that in women at high risk for arterial events, raloxifene reduced the incidence of coronary events and CVA. However, after 8 years of treatment, the incidence of cardiovascular, coronary, or cerebrovascular events did not significantly differ between the raloxifene and placebo groups. In the STAR study,^11^ the risk of CVA was similar in the raloxifene and tamoxifen groups.

### Selective estrogen receptor modulators and lipid profile

There is evidence of changes in the lipid profile with the use of SERMs. The reduction of serum total cholesterol and low-density lipoprotein cholesterol (LDL-c) levels is a consensus. However, an increase in serum triglyceride levels has also been reported. Sawada and Sato^23^ reported that tamoxifen reduced total and LDL cholesterol levels, as well as significantly increased triglycerides. Atalay et al.^24^ did not find a significant effect of tamoxifen on total cholesterol or high-density lipoprotein-cholesterol (HDL-c) but reported a borderline increase in triglycerides. Taken together, these studies suggest that although tamoxifen consistently lowers LDL-c levels, the effects on HDL-c are mild, and tamoxifen use increases serum triglyceride levels. Changes in the lipid profile associated with the use of tamoxifen are summarized in Table 1.

**Table 1:** Tamoxifen and lipid profile

Changes	Total cholesterol	LDL-c	HDL-c	Triglycerides
Tamoxifen	Reduction	Reduction	Mild alteration	Increase

LDL-c: low-density lipoprotein cholesterol; HDL-c: high-density lipoprotein cholesterol.

### Selective estrogen receptor modulators and coronary artery disease

Even after consolidation of the clinical use of tamoxifen, there is no definitive evidence of its effect on coronary artery disease (CAD). Evidence suggests a modest protective effect of tamoxifen against death from CAD. There is a controversy over the effects of SERMs on atherosclerosis and its complications (Table 2). The publication of the RUTH (Raloxifene Use for The Heart) study^21^ confirmed a neutral effect of raloxifene. Evidence available in the world literature suggests neutral effects or discrete benefits of SERM use in overall cardiovascular risk.^25^

**Table 2:** Events associated with the use of selective estrogen receptor modulators (SERMs)

Study, year	Type of study	Patients (n)	Assessed/ compared SERMs	Breast cancer	VTE	CVA	CAD
STAR, Vogel et al.^11^ 2006	Clinical trial	19,747 postmenopausal women	Tamoxifen and raloxifene	Risk reduction of 50% (*in situ* - tamoxifen and raloxifene and invasive- tamoxifen)	Increase, raloxifene < tamoxifen of 30%	Reduction (tamoxifen and raloxifene)	Increase (tamoxifen and raloxifene)
MORE, Cauley et al.^13^ 2001	Clinical trial	7,705 postmenopausal women	Raloxifene and placebo	Risk reduction of 72% after 4 years	Increase	Neutral	Neutral
CORE / Martino et al.^14^ / 2004	Clinical trial	5,213 postmenopausal women	Raloxifene and placebo	Risk reduction of 59%	Increase		
NSABP / Fisher et al.^9^ / 1998	Clinical trial	13,388 at risk for breast cancer	Tamoxifen and placebo	Risk reduction of 49%	Increase	Increase	Neutral
IBIS-1 / Cuzick et al.^8^ / 2002	Clinical trial	7,152 at risk of breast cancer	Tamoxifen and placebo	Risk reduction of 32%	Increase	Neutral	Neutral
RUTH / Barret-Connor et al.^21^ / 2006	Clinical trial	10,101 postmenopausal women	Raloxifene and placebo	Invasive cancer risk reduction of 55%	Increase of 44%	Increase of 49%	Neutral

VTE: venous thromboembolism; CAD: coronary artery disease.

In the NSABP study,^26^ 13,388 women at increased risk of breast cancer were assigned to receive tamoxifen 20 mg/day or placebo. Cardiovascular follow-up was available for 13,194 women, of which 1,048 had clinically manifest CAD. The rates of cardiovascular events were not significantly different between women receiving tamoxifen and those receiving placebo, regardless of the preexisting disease. A case-control study of women diagnosed with breast cancer found that the use of tamoxifen was not associated with a reduced risk of myocardial infarction for the observed 137 cases of myocardial infarction.^27^ Another case-control study demonstrated that women with breast cancer who received tamoxifen had a reduced risk of angina pectoris or myocardial infarction (OR = 0.4, 95%CI: 0.2-0.7) compared to patients who did not receive it.^28^

Nordenskjold et al.^29^ reported a significant reduction in mortality due to CAD in women who received 5 years vs. 2 years of tamoxifen, with a higher dose of 40 mg/day. The study carried out by the Early Breast Cancer Trialists' Collaborative Group^30^ reported a reduction in mortality from CAD in more than 15,000 women randomized to receive approximately 5 years of tamoxifen vs. placebo, although there was no statistical significance (120 vs. 132 deaths, p = 0.06).

### Selective estrogen receptor modulators and aspirin

Cancer can lead to a state of hypercoagulability, platelet abnormalities and thromboembolic events. Platelets can contribute to the metastasis process by promoting angiogenesis and by releasing the Vascular Endothelial Growth Factor (VEGF).^31^^,^^32^ the platelets and coagulation cascade components involve tumor cells, which prevents lysis by natural killer cells, allowing the spread of metastases.

Tamoxifen rapidly increases free calcium in human platelets.^33^^,^^34^ Jhonson et al.^35^ demonstrated that tamoxifen and its metabolite 4-hydroxytamoxifen altered the platelet function, with a reduction in the angiogenic and metastatic potential. Angiogenic proteins are released during the platelet activation process, and platelet deposition is observed at the tumor site.^36^ The alpha and beta forms of ER were found in the platelet membrane.^37^^,^^38^ Some studies have suggested that estradiol, as well as the tamoxifen metabolites, can increase platelet aggregation, suggesting that ER function may influence the release of intraplatelet proteins, such as VEGF and endostatin, when platelets are stimulated in the tumor environment.^39^

Holmes et al.^40^ carried out a study that evaluated the concentrations of VEGF and endostatin before and after tamoxifen or aromatase inhibitors in 30 women with breast cancer. Tamoxifen therapy resulted in increased VEGF concentrations in platelets, but no change in plasma VEGF levels. The use of aspirin attenuated the increase in the VEGF levels associated with tamoxifen and reduced serum levels of VEGF. The data from this study suggest that antiplatelet therapy may interfere with angiogenic protein levels in women treated with endocrine therapy.

Women with breast cancer who used tamoxifen and 45 days of aspirin had reduced intraplatelet VEGF levels, as well as increased serum and intraplatelet levels of the antiangiogenic factor thrombospondin-1.^41^ These changes were reversed with the aspirin discontinuation. In this study, a dose of 325 mg/day was used. Aspirin decreased the pro-angiogenic effects of tamoxifen, suggesting that antiplatelet therapy may improve tamoxifen efficacy. Cheng et al.^42^ carried out a study that showed that aspirin not only inhibits the growth of the MCF-7 RE-positive breast cancer cell line, but also has a potential function to overcome resistance to tamoxifen in MCF-7/TAM cell lines. The concomitant action of aspirin makes cells more sensitive to tamoxifen, indicating that aspirin can regulate proteins to overcome tamoxifen resistance.

The RUTH^21^ study evaluated the effects of antiplatelet therapy concomitant with the use of raloxifene regarding the risk of VTE. The increased risk of VTE with raloxifene when compared to placebo was not different between the women who used antiplatelet agents and those who did not use it.^43^ The key findings of the abovementioned studies are summarized in Table 3.

**Table 3:** Aspirin and selective estrogen receptor modulators (SERMs)

Author, year	Type of study	Patients (n)	SERMs and/or aspirin	Main Conclusions
Holmes et al.,^40^ 2008	Clinical trial	30	Tamoxifen or aromatase inhibitor + ASA	ASA attenuated the increase in VEGF associated with tamoxifen
Holmes et al.,^44^ 2010	Prospective Cohort	4,164	ASA	Reduction in the recurrence and death from breast cancer
Holmes et al.,^41^ 2013	Clinical trial	12	Tamoxifen + ASA	Reduction in VEGF and increase in TSP-1
Holmes et al.,^45^ 2014	Case-control	27,426	ASA	There is no benefit during end-stage illness
Yang et al.,^46^ 2017	Retrospective Cohort	148,739	ASA	Reduction in breast cancer risk in diabetics
Fraser et al.,^47^ 2014	Cohort	4,627	ASA	Reduction in death risk from all causes
Jhonson et al.,^48^ 2002	Prospective Cohort	27,616	ASA	Reduction in breast cancer risk
Harris et al.,^53^ 1999	Prospective Cohort	32,505	ASA/ibuprofen	Reduction in breast cancer risk
Duvernoy et al.,^43^ 2010 (RUTH Trial)	Clinical trial	10,101	Raloxifene + ASA	Did not change the risk of VTE

ASA: acetylsalicylic acid; VEGF: vascular endothelial growth factor; TSP-1: thrombospondin 1; VTE: venous thromboembolism.

### Aspirin and Cancer Prevention

A prospective observational study of 4,164 women with breast cancer showed that, among women who were alive at least 1 year after the breast cancer diagnosis, the use of aspirin was associated with a reduction in the risk of recurrence and death from breast cancer.^44^ Contrarily, another study with 27,426 women with breast cancer showed that there was no association between aspirin use and death from breast cancer.^45^

A retrospective cohort study was carried out in Taiwan with 148,739 diabetic women, of which 27,378 used aspirin at a dose ranging from 75 mg to 165 mg/day, which were compared to women who did not use aspirin. Overall, aspirin use reduced the risk of breast cancer by 18% (Hazard Ratio – HR= 0.82, 95%CI: 0.71-0.94). Specifically, a cumulative dose of aspirin > 88,900mg was observed to reduce the risk of breast cancer by 47% .^46^ A cohort in Scotland identified 4,627 women with breast cancer throughout 11 years. The use of aspirin after the diagnosis was identified in 1,035 women (22.4%). Most of them used a 75 mg dose/day. It was concluded that low-dose aspirin was associated with reduced risk of death from all causes and breast cancer.^47^ Another cohort, with 27,616 postmenopausal women, identified 938 cases of breast cancer in 6 years of follow-up, meaning a RR of 0.71 (95% CI: 0.58-0.87) for those who took aspirin at least six times a week, when compared with those who did not use the medication.^48^ Evidence from case-control and cohort studies suggest an approximately 10% reduction in the risk of breast cancer for aspirin use.^49^^,^^50^ Similar results were found with other NSAIDs and Cycloxygenase-2 inhibitors (COX-2).^51^

Rothwell et al.^52^ analyzed seven randomized trials for the regular use of aspirin with a minimum duration of 4 years to determine the effect of aspirin on the risk of death from cancer. Daily aspirin reduced death rates from several types of cancer during and after the studies. The benefit increased with treatment duration and was consistent in all the different studied populations.

Harris et al.^53^ found 393 cases of breast cancer in 32,505 patients after 5 years of follow-up. This study reported a 50% reduction in the incidence of breast cancer using ibuprofen (p < 0.01) and 40% with regular aspirin use (p < 0.05), suggesting that other NSAIDs may also be effective in breast cancer prophylaxis.

Aspirin emerged as the most likely NSAID for use in chemoprevention due to its benefits also in preventing cardiovascular events. Other NSAIDs have also been studied as adjuvants in the chemoprevention of several types of cancer, especially colorectal, breast and stomach neoplasms, although these drugs do not offer cardioprotection.^54^ Mortality reduction is more evident in colon cancer, probably in prostate and possibly also in breast neoplasms.^55^^,^^56^

## Discussion

The clinical trials mentioned in this review report an increase in VTE with the use of SERMs and, regarding cerebrovascular and coronary events, the results were discordant. The currently used treatment, consisting of chemotherapy and hormone therapy, has reduced breast cancer mortality, but morbidity and mortality are still high, with considerable side effects and high financial costs. There are great expectations regarding new treatments, with low toxicity and cost reduction.

Aspirin has been incorporated into clinical practice for over 100 years at a low cost, making it attractive as a potential adjunct treatment. The observational studies included in this review suggest a reduction in the risk of breast cancer in patients regularly taking aspirin. Randomized clinical trials are required to assess the impact of aspirin use on breast cancer prevention, whether associated with endocrine therapy or disease-free survival in breast cancer patients.

Aspirin use is a consensus for the secondary prevention of myocardial infarction and ischemic CVA in patients with pre-existing cardiovascular disease and for primary prevention in high-risk groups. Current indications for the prophylactic use of aspirin are based on cardiovascular risk, considering the side effects, especially gastrointestinal bleeding, of which incidence increases with age. Other potential benefits of using aspirin need to be proven in the context of cancer.

## Conclusion

Breast cancer is the most frequently diagnosed tumor in women worldwide, with a significant impact on morbidity and mortality. Although there are controversies in the analyzed studies, considering the possible benefits regarding breast cancer prevention and reduction in cardiovascular events, this systematic review suggests that therapy with selective estrogen receptor modulators and aspirin should be better investigated, and emphasizes the need for randomized trials. Future studies should address issues such as dose, age at the start, duration, efficacy, and safety of a clearly defined treatment regimen.
